# Adipose tissue macrophage-derived miR-690 modulates adipocyte precursor cell maintenance and adipogenesis

**DOI:** 10.1016/j.molmet.2025.102246

**Published:** 2025-09-03

**Authors:** Karina Cunha e Rocha, Breanna Tan, Julia Kempf, Cristina Medina, Varsha Beldona, Chengjia Qian, Ying Duan, Qian Xiang, Ahjin Yoo, Xiaomi Du, Amit R. Majithia, Wei Ying

**Affiliations:** 1 Department of Medicine, Division of Endocrinology & Metabolism, University of California San Diego, La Jolla, California, USA; 2 Division of Biological Sciences, University of California, San Diego, La Jolla, California, USA; 3 Chemistry Department, University of Rochester, Rochester, New York, USA

**Keywords:** Obesity, White adipose tissue, Adipose Tissue Macrophages, Adipocyte Precursor Cells, miR-690, Hyperplasia

## Abstract

Obesity is intricately linked to various metabolic diseases; however, some individuals maintain metabolic health despite being classified as obese. A critical factor underlying this paradox is the expansion of white adipose tissue (WAT), which can occur through two mechanisms: hypertrophy (the enlargement of existing fat cells) and hyperplasia (the formation of new fat cells from adipocyte precursor cells, or APCs). Hyperplasia is regarded as a healthier mode of WAT expansion, as it tends to reduce inflammation and protect against insulin resistance. Thus, interventions that promote hyperplasia over hypertrophy could improve metabolic health in obese individuals. In this study, we investigate the role of microRNA-690 (miR-690), an anti-inflammatory and insulin-sensitizing molecule, in maintaining the APC population and facilitating the healthy expansion of epididymal WAT (eWAT). Our findings indicate that in lean mice, macrophages support the APC population by transferring miR-690 to APCs. However, during obesity, the recruitment of pro-inflammatory lipid-associated macrophages (LAMs) to eWAT diminishes miR-690 delivery to APCs, impairing adipogenesis and leading to unhealthy WAT expansion. We demonstrate that strategies aimed at increasing the availability of miR-690 to APCs or mimicking its effects can restore APC functionality. Additionally, mutations in Nadk, the target of miR-690, were shown to mitigate the adverse effects of obesity on APC maintenance in eWAT. These findings suggest that targeting the miR-690-Nadk axis in APCs may provide novel therapeutic strategies to promote healthy adipose tissue expansion and protect against obesity-related metabolic diseases.

## Highlights

• In lean eWAT, macrophages help maintain adipocyte precursor cell (APC) and sustain miR-690 expression.
• Obesity depletes APCs in eWAT, reduces miR-690 expression, and shifts macrophages toward a pro-inflammatory state.
• Blocking the recruitment of pro-inflammatory macrophages to eWAT prevents APC loss during obesity.
• Enhancing miR-690 expression in APCs through mimic treatment and weight loss helps prevent APC loss during obesity.
• MiR-690 maintains APC by targeting NADK, supporting healthy adipose tissue expansion.

## 1 Introduction

Obesity is commonly associated with metabolic diseases such as insulin resistance and type 2 diabetes (T2D) [1,2]. However, individuals with obesity exhibit significant heterogeneity in cardiometabolic risk, with some classified as “metabolically healthy obese” due to their resistance to developing metabolic diseases despite excessive adiposity [[3], [4], [5], [6], [7], [8]].

A critical determinant of metabolic health in obesity is the mechanism of white adipose tissue (WAT) expansion [9]. Under calorie excess, WAT expands either through hypertrophy, where existing adipocytes increase in size, or through hyperplasia, which involves the formation of new adipocytes via the de novo differentiation of adipocyte progenitors [[9], [10], [11]]. The balance between hypertrophic expansion and hyperplasia is crucial for metabolic health, as each influences the WAT microenvironment differently. Commonly observed in obesity, hypertrophic expansion is typically unhealthy, triggering adipocytes to secrete proinflammatory cytokines, recruiting immune cells, and creating a proinflammatory microenvironment that promotes hypoxia and tissue fibrosis [9,[11], [12], [13], [14]]. Conversely, metabolically healthy obesity is characterized by beneficial adipose tissue expansion driven by hyperplasia, where new, smaller, and more insulin-sensitive adipocytes derived from adipocyte precursor cells (APCs), which consist of adipose stem and progenitor cells (ASPCs) committed to adipocyte lineage differentiation [9,11,[15], [16], [17], [18]]. Therefore, the ability of APCs to differentiate into adipocytes, a process known as adipogenesis, is essential for promoting healthy adipose tissue expansion [9,19,20]. Thus, developing strategies that promote APC differentiation could protect against metabolic diseases associated with obesity.

It is well established that during obesity, the expansion of WAT is accompanied by the recruitment of adipose tissue macrophages (ATMs) derived from circulating monocytes, predominantly differentiating into pro-inflammatory macrophages [21,22]. Furthermore, a reduced M1-like/M2-like ATM ratio has been associated with healthy WAT expansion [23]. Our group has previously demonstrated that ATMs can modulate insulin sensitivity and inflammation through the release of microRNA (miRNA)-containing extracellular vesicles (EVs) [24]. Notably, miR-690, enriched in anti-inflammatory M2-like macrophages, exerts insulin-sensitizing and anti-inflammatory effects via modulation of NAD + metabolism [25]. Given that macrophages have been identified as the primary source of miR-690 in the liver [26]*,* and that NAD + availability regulates adipogenesis [27], we hypothesized that in the epididymal white adipose tissue (eWAT), ATM-derived miR-690 could influence the APC population, thereby modulating adipogenesis and eWAT expansion.

In this study, we demonstrate that in lean mice, anti-inflammatory macrophages serve as the primary source of miR-690, which is delivered to APCs in the eWAT. This ATM-derived miR-690 delivery is key for maintaining the APC population and ensuring proper adipogenesis. However, during obesity, the recruitment of pro-inflammatory lipid-associated macrophages leads to reduced miR-690 levels in ATMs, impairing miR-690 delivery to APCs. Consequently, this disruption leads to a reduction in the APC population, compromising adipogenesis. Importantly, we found that APC-specific miR-690 overexpression or depletion of a known miR-690 target, *Nadk*, protected against obesity-induced reduction in APC population and preserved adipogenesis. Additionally, weight loss restored anti-inflammatory ATMs populations in the eWAT, re-establishing ATM-derived miR-690 delivery and consequently increasing miR-690 levels in APCs, ultimately rescuing the APC population. These findings suggest that enhancing the ATM-derived miR-690-Nadk regulatory axis in APCs represents a promising therapeutic strategy to promote healthy adipose tissue expansion.

## 2 Results

### 2.1 miR-690-mediated crosstalk between ATMs and APCs maintains the APC population

**Figure 1:**
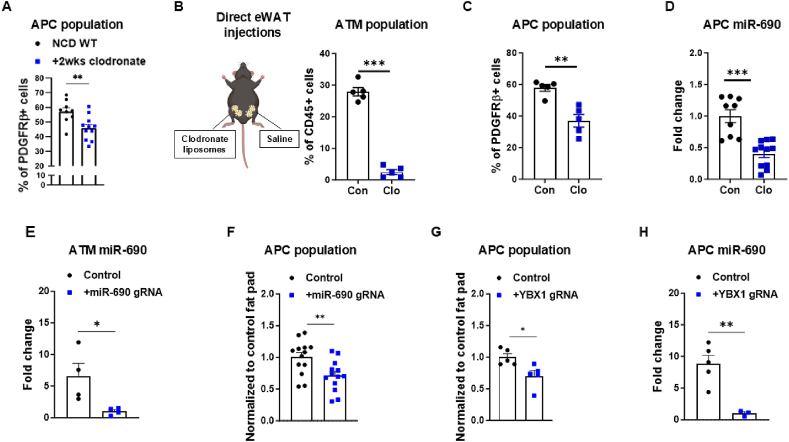
**Adipose tissue macrophages maintain adipocyte precursor cells through miR-690 secretion.** (**A**) Adipocyte precursor cell (APC) population in the epididymal white adipose tissue (eWAT) of 10–12 week-old normal chow diet-fed male mice after 2 weeks of intraperitoneal injection of clodronate liposomes. (**B**) Direct injections of clodronate liposomes (Clo) were performed in one pad of eWAT of lean WT mice, while the other pad was treated with saline. (**B**) After 4 days, the population of adipose tissue macrophages (ATMs) and (**C**) APCs were analyzed by FACS assays, and (**D**) APC miR-690 abundance was quantified by qPCR assays. To obtain macrophage-specific miR-690 repression in eWAT, direct injections of lentivirus carrying gRNA-miR-690 or empty vectors (Control) were performed in the eWAT of lean LysMCre^+^Cas9^f^^lox^^/f^^lox^ male mice. (**E**) Seven days later, the abundance of miR-690 in ATMs and (**F**) the APC population were analyzed. Similarly, direct injections of lentivirus carrying Ybx1 gRNA or empty vectors (Control) into the eWAT of lean LysMCre^+^Cas9^f^^lox^^/f^^lox^ male mice were performed. (**G**) The effect of macrophage-specific YBX1 repression on APC population and (**H**) miR-690 abundance in eWAT was measured seven days after injections. Data are presented as mean ± SEM. ^∗^*P* ≤ 0.05, ^∗∗^*P* ≤ 0.01, ^∗∗∗^*P* ≤ 0.001, Student's t-test. *n* = 8 (NCD WT) and *n* = 11 (+2wks clodronate) for (**A**); *n* = 5 mice/group for (**B–C**); *n* = 9 (control) and *n* = 11 (clodronate) for (**D**); *n* = 4 mice/group for (**E**); *n* = 13 mice/group for (**F**); *n* = 5 mice/group for (**G**); *n* = 5 (control) and *n* = 3 (+YBX1 gRNA) for (**H**).

Previous studies suggest that adipose tissue macrophages play a critical role in mediating the interactions between neighboring cells in adipose tissues. We first assessed the role of macrophages in maintaining adipocyte precursor cells. To achieve this, we utilized lean wild-type (WT) male C57BL/6 mice and administered clodronate liposomes intraperitoneally to deplete the macrophage population [28]. As expected, two weeks of intraperitoneal (i.p.) clodronate liposome treatment (0.35mg/mouse; twice per week) effectively depleted most of the ATMs in the eWAT (Fig. S1A). Supporting our hypothesis that ATMs are important for APC maintenance, depletion of the macrophage population in the eWAT was accompanied by a reduction in the APC population in the eWAT (Figure 1A&S1B). However, it is important to note a limitation: i.p. injection of clodronate liposomes leads to systemic macrophage depletion, likely inducing pan-effects on eWAT APC phenotypes. To overcome this, we developed a localized delivery strategy by injecting clodronate liposomes directly into the eWAT. One concern with this approach was the potential leakage of liposomes into the circulation, potentially reaching other tissues. To evaluate this, we conjugated the liposomes with PKH26, a red fluorescent dye, and injected them directly into one eWAT pad of lean WT mice, with saline being injected into the other eWAT pad as a control. After 24 h, flow cytometry analysis revealed minimal leakage of the clodronate liposomes from the injection site (Fig. S1C). Encouraged by these results, we proceeded to inject lean WT animals directly into one eWAT fat pad with clodronate liposomes and saline into the contralateral pad as a control (Fig. 1B). Consistent with previous i.p. treatments, direct clodronate liposome injection led to a depletion of the eWAT macrophage population and resulted in a concomitant reduction in the APC population within the same fat pad (Fig. 1C).

Our previous work has demonstrated that in lean mice, tissue-resident macrophages mediate neighboring cell functions through the secretion of miR690, which is an insulin sensitizer and anti-inflammatory microRNA [25,26]. Further highlighting the potential for crosstalk, macrophage depletion decreased levels of miR-690 in APCs within the eWAT (Fig. 1D). These findings underscore the significance of ATMs in maintaining the APC population and suggest a potential mechanism involving miR-690 delivery from ATMs to APCs.

To deepen our understanding of the role of ATMs in regulating APC miR-690 expression, we implemented a macrophage-specific miR-690 knockout strategy using CRISPR/Cas9-loxP-mediated genetic modification. This approach involved crossbreeding LysMCre-expressing mice with LSL-nuclease-activated Cas9 knock-in mice, which carry a floxed-stop cassette hindering Cas9 expression [29,30]*.* Using our direct eWAT injection technique, we induced ATM-specific miR-690 knockout in one eWAT pad by delivering a lentivirus carrying gRNA-miR-690 to LysMCre^+^Cas9^+^ mice, while the contralateral pad served as the control and received a control lentivirus containing empty vectors. Lentiviral leakage from the injection site was minimal, as demonstrated by only a few PKH26-labeled particles detected in the control eWAT (Fig. S1D). Validating our model, ATM miR-690 expression was reduced in the pads injected with gRNA-miR-690 (Fig. 1E). Importantly, there was no significance difference in the macrophage population between the eWAT pads injected with either miR-690 gRNA or the control lentivirus (Fig. S1E). As a result of the specific depletion of miR-690 in eWAT ATMs, we observed a reduction in the APC population (Fig. 1F).

Having identified ATMs as a potential source of miR-690 for APCs, we next investigated the mechanisms underlying miR-690 delivery. Previous work from our group demonstrated a similar finding in the liver, where resident macrophages were shown to be the main source of miR-690 for neighboring hepatic cells, with extracellular vesicles (EVs) serving as the primary delivery mechanism [26]*.* To investigate whether EVs also mediate miR-690 transfer between ATMs and APCs, we generated an ATM-specific knockout for Ybx1, a recognized protein responsible for sorting miRNAs into EVs [31,32].This was achieved by injecting Ybx1-gRNA into one eWAT of LysMCre^+^Cas9^+^ mice, while the contralateral pad received a control lentivirus carrying empty vectors. Although this approach has been used in similar contexts to generate EVs selectively depleted of miRNAs [32], we validated our model by confirming efficient Ybx1 knockdown in ATMs and demonstrating trend toward reduced miR-690 abundance in ATM-derived EVs from the Ybx1 knockdown group compared with controls (Figure 1F&G). Importantly, EV particle concentration and size distribution did not differ significantly between groups (Fig. S1H&I). Interestingly, the reduction of miRNAs in ATM-derived EVs not only led to a decrease in the APC population (Fig. 1G) but also reduced miR-690 expression within APCs (Fig. 1H). These findings highlight the importance of ATM-derived miRNAs, particularly miR-690, in supporting APC maintenance and underscore EVs as a key vehicle for this ATM-APC communication.

### 2.2 Recruited proinflammatory macrophages drive obesity-related disruption of the ATM and APC population

**Figure 2:**
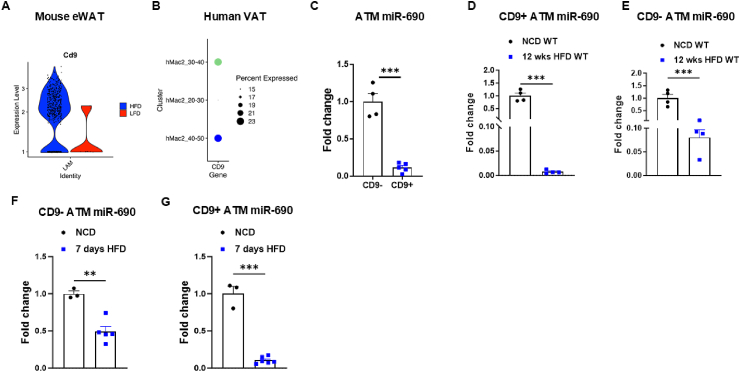
**Adipose tissue macrophage phenotypes in obesity.** (**A**) In silico analysis showing the abundance of CD9 in lipid-associated macrophages (LAMs) in the epididymal white adipose tissue (eWAT) of mice or (**B**) visceral adipose tissue (VAT) of humans. (**C**) miR-690 abundance in CD9-vs. CD9+ adipose tissue macrophages (ATMs) in the eWAT of lean WT mice. MiR-690 expression in (**D**) CD9+ or (**E**) CD9- ATMs in the eWAT after 12 weeks of high-fat diet (HFD) feeding. miR-690 abundance in (**F**) CD9-or (**G**) CD9+ ATMs in the eWAT after 7 days of HFD feeding. Data are presented as mean ± SEM. ^∗∗^*P* ≤ 0.01, ^∗∗∗^*P* ≤ 0.001, Student's t-test. *n* = 6 mice/group for (**A**); *n* = 10 individuals total for (**B**); *n* = 4 (CD9^-^) and *n* = 5 (CD9^+^) for (**C**); *n* = 4 mice/group for (**D–E**); *n* = 3 (NCD) and *n* = 5–6 (7-day HFD) for (**F–G**).

While we have established the role of ATMs in maintaining the APC population and miR-690 levels in the eWAT, it is important to note that our analyses thus far have primarily focused on lean eWAT, which is typically enriched with anti-inflammatory ATMs [33]. To expand on this, we next investigated whether distinct macrophage populations have different effects on APC maintenance. At the adipose tissue level, obesity is characterized by a transition from anti-inflammatory to pro-inflammatory macrophages, accompanied by an increase in monocyte/macrophage recruitment [21,22]. Recently, a pro-inflammatory macrophage subtype population undergoing significant expansion during obesity has been identified in both human and mouse adipose tissue [34]. These macrophages, known as lipid-associated macrophages (LAMs), express markers such as CD9, TREM2, and GPNMB [34,35]. Given this, we sought to investigate whether the transition from anti-inflammatory to pro-inflammatory macrophage subtypes, particularly the recruitment of LAMs to adipose tissue induced by high-fat diet (HFD) feeding and obesity, could influence the APC population. Initially, we utilized available single-nucleus RNA sequencing (snRNA-seq) data from both mouse and human visceral adipose tissue (VAT) to confirm whether obesity leads to an increase in LAMs [36,37]. In mouse VAT, we observed elevated expression of CD9, a key LAM marker, within the LAM population of obese mice compared to lean mice [36] (Figure 2A). A similar trend was observed in mouse VAT and human omental VAT, as reported by Emont et al., 2022, in which mice fed with chow or HFD and human subjects with varying ranges of BMI (20–30, 30–40, and 40–50) where profiled [37]. This dataset included three macrophage subpopulations in humans (hMac1, hMac2, and hMac3) and four in mice (mMac4, mMac3, mMac2 and mMac1), among which hMac2 and mMac3 exhibited high expression of major LAM markers, suggesting that these population corresponds to LAMs (Figure 2A&B). When we analyzed the expression of CD9 within the LAM population, we found that LAMs from subjects with higher BMI exhibited increased expression of CD9 compared to those with a BMI ranging from 20 to 30 (Fig. 2B). Next, we investigated the relationship between LAM identity and miR-690 expression. In lean mouse eWAT, CD9+ ATMs expressed significantly lower levels of miR-690 than CD9- ATMs (Fig. 2C). After 12 weeks of HFD feeding, miR-690 was greatly repressed in both CD9-and CD9+ ATMs (Figure 2D,E, S2C). We also found that a short period (7 days) of HFD feeding resulted in a rapid reduction in miR-690 levels in eWAT ATMs (Figure 2F,G, S2D&E).

**Figure 3:**
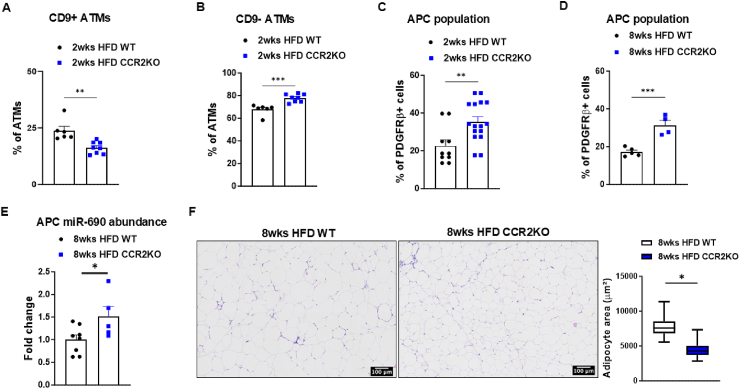
**Blocking lipid-associated CD9+ macrophage recruitment attenuates adipocyte precursor cell loss in obesity.** The populations of (**A**) CD9+ adipose tissue macrophages (ATMs), (**B**) CD9- ATMs, and (**C**) adipocyte precursor cells (APCs) in the eWAT of WT vs. CCR2KO mice after 2 weeks of high-fat diet (HFD) feeding. (**D**) APC population, (**E**) miR-690 levels in APCs, and (**F**) the adipocyte size in the eWAT of WT vs. CCR2KO mice after 8 weeks of HFD feeding. Data are presented as mean ± SEM. ^∗^*P* ≤ 0.05, ^∗∗^*P* ≤ 0.01, ^∗∗∗^*P* ≤ 0.001, Student's t-test. Scale bar, 100 μm. *n* = 6 (2wks HFD WT) and *n* = 8 (2wks HFD CCR2KO) for (**A–B**); *n* = 10 (2wks HFD WT) and *n* = 16 (2wks HFD CCR2KO) for (**C**); *n* = 5 (8wks HFD WT) and *n* = 4 (8wks HFD CCR2KO) for (**D, F**); *n* = 8 (8wks HFD WT) and *n* = 5 (8wks HFD CCR2KO) for (**E**).

LAMs are typically recruited from circulating monocytes and are commonly found surrounding crown-like structures surrounding lipid-laden and cell death-prone adipocytes [34]. CCR2 (CC chemokine receptor 2) is recognized as an important chemokine receptor crucial for the recruitment of monocyte-derived macrophages from peripheral blood [[38], [39], [40]]. To investigate the impact of recruited macrophages on the eWAT APC population under chow diet conditions, as well as during both short-term and long-term HFD feeding, we conducted experiments using whole-body CCR2 knockout (CCR2KO) mice. As a baseline control, we compared NCD-fed CCR2KO mice with age-matched wild-type (WT) mice. No differences were observed between the groups in terms of body weight, eWAT weight, liver weight, ATM and APC populations, or adipocyte morphology (Fig. S3A–H). In the short term, WT and CCR2KO mice were fed HFD for two weeks. Despite showing no differences in body weight, eWAT weight, or total ATM population, CCR2KO mice exhibited a reduced population of CD9+ ATMs and a corresponding increase in CD9- ATM population (Figure 3A,B, S3I-K). CCR2KO mice also exhibited a larger APCs population in the eWAT after two weeks of HFD feeding (Fig. 3C). Similar to what was observed in the short-term, CCR2KO mice presented a higher APC population in the eWAT after 8 weeks of HFD feeding compared to their wild-type counterparts (Fig. 3D). Furthermore, APCs isolated from the eWAT of CCR2KO mice after 8 weeks of HFD feeding showed elevated miR-690 levels relative to obese WT mice (Fig. 3E). Additionally, the absence of macrophage recruitment mitigated several known effects of HFD feeding, including body weight gain, increased eWAT and liver weights, and expansion of the ATM population in the eWAT (Fig. S3L-O). Corroborating with these findings, CCR2KO mice presented smaller eWAT adipocytes after 8 weeks of HFD feeding (Fig. 3F).

### 2.3 Obesity disrupts miR-690-mediated ATM-APC crosstalk and impacts APC population maintenance

**Figure 4:**
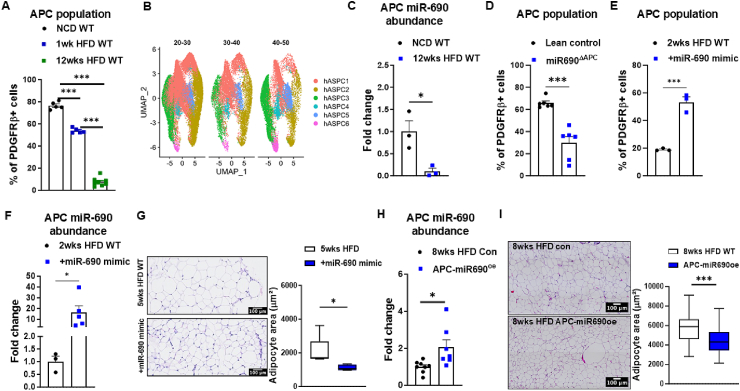
**The importance of miR-690 on adipocyte precursor cell maintenance.** (**A**) Adipocyte precursor cell **(**APC) population in epididymal white adipose tissue (eWAT) of mice during obesity progression promoted by high-fat diet (HFD) feeding. (**B**) Human snRNA-seq data ilustrates how adipose stem and progenitor cells (hASPC) populations vary across different body mass index (BMI) ranges. (**C**) APC miR-690 abundance in eWAT of lean vs. 12-week HFD-fed WT mice. In lean PDGFRbCre^+^Cas9^+^ mice, direct eWAT injections with lentivirus carrying miR-690gRNA were performed to induce APC-specific miR-690 deletion, while the control group received empty vector injections. (**D**) The effect of APC-specific miR-690 deletion on the APC population in the eWAT of lean mice. (**E**) APC population and (**F**) miR-690 expression in eWAT of HFD-fed WT mice after 2 weeks of intraperitoneal (i.p.) injections with miR-690 mimic. (**G**) HFD-fed control mice were treated with scramble microRNA mimic. Adipocyte size in the eWAT of HFD WT mice after 5 weeks of i.p. injections with miR-690 mimic. (**H**) APC miR-690 abundance and (**I**) adipocyte size in the eWAT of 8 weeks HFD-fed PDGFRbCre^+^dCas9^+^ mice after direct eWAT injections with lentivirus carrying miR-690gRNA and the VP64 activator to promote APC-specific miR-690 overexpression. Control PDGFRbCre^+^dCas9^+^ mice were treated with lentivirus-carrying empty vectors and fed HFD for 8 weeks. Data are presented as mean ± SEM. ^∗^*P* ≤ 0.05, ^∗∗∗^*P* ≤ 0.001, Student's t-test. Scale bar, 100 μm. *n* = 5 (NCD WT), *n* = 5 (1wk HFD WT), and *n* = 8 (12wks HFD WT) for (**A**); *n* = 10 individuals total for (**B**); *n* = 3 mice/group for (**C**); *n* = 6 mice/group for (**D**); *n* = 3 mice/group for (**E**); *n* = 3 (2wks HFD WT) and *n* = 5 (+miR-690 mimic) for (**F**); *n* = 7 mice/group for (**G**); *n* = 8 (8wks HFD control) and *n* = 7 (APC-miR-690^oe^) for (**H**); *n* = 9 mice/group for (**I**).

Consistent with previous reports, we observed that the loss of APCs was accompanied by the development of obesity (Figure 4A). Similarly, analysis of human snRNA-seq data yielded comparable findings. Utilizing data from Emont et al., 2022 [37] six distinct human adipose stem and progenitor cell (hASPC) populations were identified, with hASPC1, hASPC2, and hASPC3 being delineated as early adipocyte progenitors, akin to the APC population used in this study, which was first characterized by Hepler et al., 2018 [18]. To identify the APC population, we focused on clusters with high expression of mature adipocyte markers, including *PPARG*, *CD36*, and *FABP4*, and low expression of *CD9* (Fig. S4A). Higher BMI levels were associated with a decrease in most ASPC populations, particularly in the hASPC1 and hASPC2 clusters (Fig. 4B). With the observation that HFD feeding increases the presence of recruited pro-inflammatory macrophages in the eWAT, negatively affecting the maintenance of the APC population, we then aimed to understand how obesity influences the communication mediated by miR-690 between ATMs and APCs. Since obese mice had lower expression of miR-690 in the APCs of eWAT (Fig. 4C), we next assessed the role of miR-690 in APC phenotypes. Intriguingly, when we directly injected miR-690-gRNA into the eWAT of lean PDGFRbCre^+^Cas9^+^ mice to create APC-specific miR-690KO, we observed that the depletion of miR-690 in APCs directly affected the APC population, resulting in a decreased population compared to WT mice (Fig. 4D, S4B). To further explore the mechanism by which HFD and miR-690 influence APC maintenance, we analyzed snRNA-seq data from mouse eWAT to evaluate markers of proliferation and survival in adipose stem and progenitor cells (ASPCs) [37]. Mki67 expression was limited across all ASPC populations, suggesting low baseline proliferative activity, while Bcl2 expression was more broadly observed (Fig. S5A–C). In the APC subset, HFD feeding was associated with increased expression of pro-apoptotic markers (Bak1 and Cycs) and stable Bcl2 levels, suggesting a shift toward apoptosis (Fig. S5D–F). To test whether miR-690 protects APCs from cell death, we directly injected miR-690 mimic or control liposomes into opposite eWAT fat pads of lean mice and analyzed Annexin V staining by FACS one week after HFD feeding. miR-690-treated APCs exhibited significantly reduced Annexin V signal compared to controls, indicating lower levels of apoptosis (Fig. S5G). Together, these findings suggest that HFD impairs APC maintenance by increasing apoptosis, and that miR-690 can preserve the APC population by preventing cell death. Having identified apoptosis as a contributing factor to APC loss during obesity, and a potential target of miR-690, we next assessed whether treatment with miR-690 mimic encapsulated in liposomes during HFD feeding would mitigate the depletion of the APC population. To confirm whether i.p. injections effectively delivered the miR-690 mimic to the eWAT, we treated lean WT animals with miR-690 mimic liposomes. After 2 weeks of HFD feeding and mimic treatment, animals that received the miR-690 mimic exhibited a higher population of APCs in the eWAT (Fig. 4E), and the APCs expressed more miR-690 compared to the scrambled RNA mimic control group (Fig. 4F). In another cohort of HFD-fed WT mice, after 5 weeks of HFD feeding with miR-690 mimic liposomes i.p. treatment, mice exhibited smaller adipocytes in the eWAT compared to the animals that received scrambled RNA mimic, suggesting that the maintenance of APCs caused by miR-690 mimic treatment promoted eWAT hyperplasia (Fig. 4G). However, it is important to point out possible whole-body effects of miR-690 mimic treatment, once we showed that animals treated with miR-690 mimic for 5 weeks while on HFD feeding have lower body weight and lower eWAT weight (Figure 4C&D).

To overcome this possible limitation, we established an APC-specific miR-690 overexpression model using a deactivated Cas9 (dCas9) transgenic mouse model. For that, we crossbreed PDGFRbCre-expressing mice with the LSL-nuclease-dCas9 knock-in mice, which have a floxed-STOP cassette blocking expression of dCas9 fused with the SunTag domain [[41], [42], [43], [44], [45]]. To activate the expression of miR-690 in APCs, we injected lentivirus carrying gRNA-miR-690 and vP64 into one eWAT fat pad of PDGFRbCre^+^dCas9^+^ mice, while the other eWAT fat pad was injected with lentivirus carrying only vP64. Mice were fed with a HFD for 8 weeks after miR-690 overexpression in APCs (APC-miR-690^oe^). After 8 weeks of HFD feeding, miR-690 expression remained elevated in APCs from the APC-miR-690^oe^ fat pad compared to the control fat pad, indicating that miR-690 expression was sustained in eWAT even after, indicating that miR-690 overexpression was sustained in the APCs of the eWAT even after HFD feeding (Fig. 4H). Consequently, we observed smaller adipocytes in the eWAT APC-miR-690^oe^ fat pad, mirroring the findings observed during miR-690 mimic treatment (Fig. 4I). To assess how miR-690-mediated changes in adipocyte size affect metabolism, we measured pAKT levels in eWAT as a marker of insulin sensitivity. The APC-miR-690oe fat pad exhibited increased pAKT levels compared to the control fat pad, suggesting enhanced insulin signaling (Fig. S4E–G). In contrast, PLIN1 levels, a marker of mature adipocytes, remained consistent between the two fat pads (Fig. S4E–G). Thus, these results suggest that miR-690 promotes hyperplasia during eWAT expansion, thereby contributing to improved insulin sensitivity. Hence, maintaining miR-690 levels during obesity may help alleviate hypertrophic eWAT expansion and protect against insulin resistance.

### 2.4 NAD kinase depletion promotes APC maintenance in obesity

**Figure 5:**
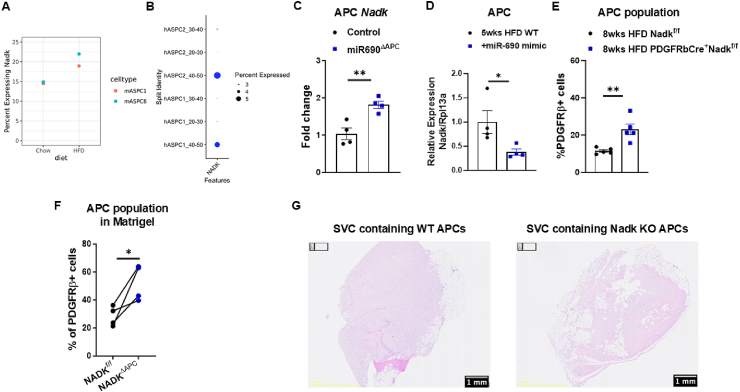
**Nadk mutation promotes adipocyte precursor cell maintenance in obesity**. Expression levels of *Nadk* in adipocyte precursor cells (APCs) from visceral fat tissues of (**A**) mice and (**B**) humans. (**C**) APC *Nadk* abundance after APC-specific miR-690 deletion induced by direct eWAT injections of lentivirus carrying miR-690gRNA into PDGFRbCre^+^Cas9^+^ mice, while the control group was injected with empty vectors. (**D**) *Nadk* abundance in the APCs isolated from the eWAT of HFD-fed WT mice after 5 weeks of intraperitoneal injections with miR-690 mimic, while HFD-fed control mice were treated with scramble microRNA mimic. (**E**) APC population in eWAT of 8 weeks HFD-fed Nadk^f^^lox^^/f^^lox^ vs. PDGFRbCre^+^Nadk^f^^lox^^/f^^lox^ mice. (**F**) After 2 weeks of Matrigel implantation in HFD-fed WT recipient mice, the APC population in the Matrigel was examined by FACS analysis. (**G**) Matrigel histological hematoxylin and eosin (H&E) analysis showing adipocyte phenotypes after 4 weeks implantation in HFD-fed mice. Data are presented as mean ± SEM. ^∗^*P* ≤ 0.05, ^∗∗^*P* ≤ 0.01, Student's t-test. Scale bar, 1 mm. *n* = 6 mice/group for (**A**); *n* = 10 individuals total for (**B**); *n* = 4 mice/group for (**C–D**); *n* = 5 mice/group for (**E**); *n* = 4 mice/group for (**F–G**).

With our results highlighting the significance of miR-690 on the crosstalk between ATMs and APCs for the regulation of the eWAT APC population, we explored possible mechanisms by which miR-690 operates within APCs. Previous studies from our group have identified *Nadk*, a gene encoding NAD kinase, as a target repressed by miR-690 [25]. Knowing that 12 weeks of HFD were able to reduce miR-690 in the APC (Fig. 4C), we subsequently examined *Nadk* expression in APCs of obese versus lean mice using the available snRNA-seq data from Emont et al., 2022 [37]. Consistent with our expectations, after 19 weeks of HFD feeding, *Nadk* expression was expressed in more cells in the two mASPC clusters representing the previously characterized APC population compared to lean animals (Figure 5A). These clusters exhibit high expression of adipocyte-related genes and low expression of *Cd9* and *Ly6c*, suggesting their classification as APCs (Fig. S6A). The increased *Nadk* levels in APCs suggest that lower miR-690 levels during HFD feeding lead to reduced repression of *Nadk* expression. Similarly, in humans with higher BMI ranges (30–40 and 40–50), the APC population (hASPC1 and hASPC2 clusters) exhibited elevated expression of *Nadk* compared to individuals with BMI ranging from 20 to 30 (Fig. 5B). We also found that miR-690KO animals exhibited elevated *Nadk* expression compared to WT mice, whereas miR-690 mimic treatment repressed *Nadk* levels in eWAT APCs of HFD-fed mice (Figure 5C,D).

Considering the regulatory role of miR-690 on *Nadk* levels, we established an APC-specific *Nadk* knockout (APC-NadkKO) mouse model by breeding PDGFRbCre-expressing mice with *Nadk* floxed mice. PDGFRbCre^+^Nadk^flox/flox^ mice and their Nadk^flox/flox^ littermates were subjected to HFD for 8 weeks. We confirmed *Nadk* repression in APCs of PDGFRbCre^+^Nadk^flox/flox^ after 8 weeks of HFD feeding (Fig. S6B). Despite no differences in body weight or eWAT weight, *Nadk* mutation in APCs attenuated HFD-induced APC loss in the eWAT (Fig. 5E, S6C-E).

After observing the impact of Nadk on the APC population, we further investigated how important Nadk is for APC-derived adipogenesis. For this purpose, we isolated the stromal vascular cells (SVCs, containing APCs) from the eWAT of both lean PDGFRbCre+Nadk^flox/flox^ (APC-NadkKO) mice and their Nadk^flox/flox^ littermates. Subsequently, we combined the SVCs with a Matrigel matrix (106 cells/400ul Matrigel) and implanted the WT or APC-NadkKO SVC–Matrigel complex into two different peritoneal areas of the same lean WT recipient [46]. Following the SVC-matrigel implantation, we induced adipogenesis by subjecting the recipients to an HFD regimen. After 2 weeks of HFD feeding, flow cytometric analysis showed that the Matrigel containing APC-NadkKO SVCs exhibited a greater APC population compared to the WT SVC implantations (Fig. 5F). Another cohort of recipients implanted with SVC Matrigel was fed HFD for 4 weeks to further induce adipogenesis. Consistent with previous studies, we observed the formation of adipocytes in the Matrigel after 4 weeks HFD feeding (Fig. 5G). This increased APC population corresponded with enhanced adipocyte presence in the histological analysis of APC-NadkKO SVC–Matrigel implantation relative to the WT implantation, suggesting that the downregulation of Nadk is crucial for APC maintenance and further adipogenesis (Fig. 5G). We also addressed the potential concern of adipocyte contamination in the implanted SVCs, as it could confound our analysis. To assess this possibility, we isolated SVCs and implanted them with Matrigel into lean WT mice. After 24 h of HFD feeding, we collected the Matrigel implants for histological analysis. No adipocytes were detected in the implants at this early time point, indicating that the adipocytes observed after 4 weeks of HFD in our experiments originated from the differentiation of APCs (Fig. S6F). To further support the role of the miR-690–Nadk axis in adipogenesis, we performed in vitro experiments using 3T3-L1 preadipocytes. Cells were transfected with a miR-690 mimic, siRNA targeting Nadk, a combination of both, or scrambled control RNA prior to the induction of adipocyte differentiation. Consistent with the in vivo findings, all treatment groups (miR-690 mimic, siRNA-*Nadk*, and the combination) showed a shift toward smaller lipid droplets compared with controls (Fig. S6G–J). The reduction in mean lipid droplet size was primarily attributable to a lower frequency of large lipid droplets after 7 days of differentiation (Fig. S6G–J). Additionally, all treated groups showed reduced triglyceride content compared with controls (Fig. S6K). These findings support our hypothesis that miR-690, potentially through downregulation of *Nadk*, promotes adipogenesis and favors the formation of smaller lipid droplets.

### 2.5 Weight loss is accompanied by APC recovery

**Figure 6:**
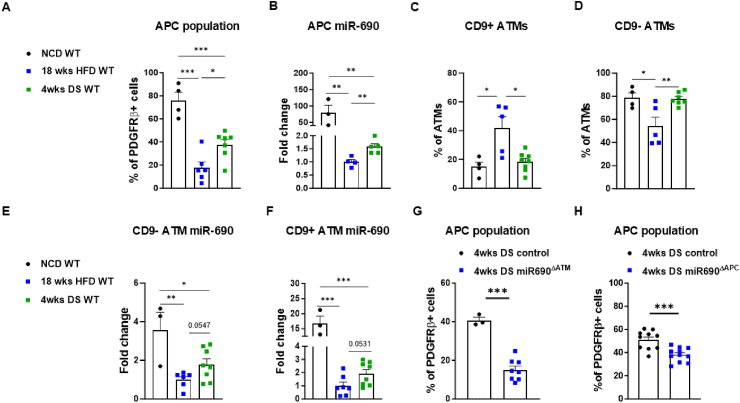
**Weight loss recovers adipocyte precursor cell maintenance in visceral adipose tissues**. (**A**) Adipocyte precursor cell (APC) population and its (**B**) miR-690 expression, (C) CD9+ adipose tissue macrophages (ATMs), (D) CD9- ATMs, and their respective (**E&F**) miR-690 abundance in the epididymal white adipose tissue (eWAT) after 4 weeks of diets-switch (DS) from a 14 weeks high-fat diet (HFD) to a normal chow diet (NCD). (**G**) Effect of macrophage-specific or (**H**) APC-specific miR-690 deletion on the APC population in eWAT of DS mice. miR-690 depletion in macrophages and APCs was achieved by direct eWAT injections of miR-690gRNA into LysMCre^+^Cas9^+^ or PDGFRbCre^+^Cas9^+^ mice, respectively, while control mice received empty vector injections. Data are presented as mean ± SEM. ^∗^*P* ≤ 0.05, ^∗∗^*P* ≤ 0.01, ^∗∗∗^*P* ≤ 0.001, Student's t-test. *n* = 3–4 (NCD WT), *n* = 4–7 (18wks HFD WT), and *n* = 5–8 (4wks DS WT) for (**A–F**); *n* = 3 (4wks DS control) and *n* = 8 (4wks DS miR-690^▲^ATM) for (**G**); *n* = 10 (4wks DS control) and *n* = 11 (4wks DS miR-690^▲APC^) for (**H**).

Previous studies have suggested that weight loss decreases ATM proinflammatory response to a state similar to that observed in lean mice [47]. Based on these findings, we aimed to investigate whether weight loss could serve as a strategy for restoring the anti-inflammatory ATM population and its miR-690 expression, thus re-establishing the ATM-APC miR-690 crosstalk that was disrupted during obesity. Weight loss was induced by subjecting WT mice to a HFD for 14 weeks, followed by a switch to a NCD for 4 weeks (diet switch, DS). As anticipated, the DS resulted in weight loss in WT mice, reducing their eWAT weight to levels comparable to those of lean mice maintained on a NCD for 18 weeks (Figure 7A&B). Analysis of the APC population in the eWAT revealed that 4 weeks of DS partially restored the depleted APC population observed after 18 weeks of HFD feeding (Figure 6A). Additionally, miR-690 expression in the APCs was also partially recovered following DS (Fig. 6B). Interestingly, the effects of the DS appeared to be more prominent in the eWAT ATM population, as shown by the levels of both CD9+ and CD9- ATM completely recovered to the levels observed in lean mice (Figure 6C,D). Similar to the findings in the APCs, miR-690 expression in both CD9+ and CD9- ATMs was partially recovered after 4 weeks of DS (Figure 6E,F).

Next, we sought to investigate the role of miR-690 in the observed effects of DS on APC and ATM phenotypes. To accomplish this, we fed LysMCre^+^Cas9^+^ and PDGFRbCre^+^Cas9^+^ mice a HFD for 14 weeks. Subsequently, we administered direct injections of miR-690-gRNA into both eWAT pads to generate ATM- and APC-specific miR-690 knockout mice, respectively, while either LysMCre^+^Cas9^+^ or PDGFRbCre^+^Cas9^+^ mice were injected with lentivirus containing empty vectors to serve as the controls. After both eWAT pads were directly injected with lentivirus harboring miR-690-gRNA, we switched the animals to an NCD for 4 weeks. Our results show that miR-690 in both ATMs and APCs is important for DS-induced APC recovery, as evidenced by less APC population in the eWAT with either macrophage- or APC-specific miR-690 mutation (Figure 6G and H, S7C-E).

## 3 Discussion

This study demonstrated the critical role of tissue-resident macrophages in mediating APC maintenance and adipogenesis. In a lean/healthy condition, ATMs support APC maintenance through miR-690 secretion. In contrast, depletion of either ATMs or ATM miR-690 caused loss of APCs in fat tissues of lean mice. In addition, the removal of EV microRNA cargos impaired the ability of ATMs to maintain the APC population. APC-specific reduction of miR-690 abundance resulted in decreased APC number in lean mouse eWAT. In obesity, there was a significant increase in lipid-associated CD9+ macrophages, along with reduced miR-690 levels in both ATMs and APCs, resulting in a great reduction in the APC population. However, blocking macrophage recruitment using CCR2-deficient mice attenuated obesity-induced miR-690 repression and APC loss in eWAT, leading to a higher number of small adipocytes. Likewise, miR-690 mimic treatment or APC-specific miR-690 overexpression improved APC maintenance in obesity, increasing the proportion of small adipocytes in eWAT and, consequently, promoting insulin sensitivity. Transcriptomics analysis of cell survival pathways showed that HFD promotes a pro-apoptotic shift in APCs, while miR-690 limits APC loss by protecting against apoptosis. The role of a known miR-690 target, Nadk, in this process was demonstrated using APC-specific *Nadk* knockout mice and Matrigel implantation assays. We also found that weight loss restored miR-690 levels in ATMs and APCs, concomitant with recovery of APC population in eWAT. However, when miR-690 was specifically mutated in either macrophages or APCs, weight loss failed to restore the APC population.

Tissue-resident macrophages play a crucial role in tissue remodeling and repairing by orchestrating the niche for neighboring stem cell activities during tissue injury [48]. While macrophages are a dominant immune cell type in adipose tissue, little is known about the importance of ATMs on APC phenotypes. Our study found that the removal of tissue-resident macrophages resulted in a significant reduction in APC number in visceral fat tissues of lean mice. In response to obesity, macrophage recruitment increases, and ATMs undergo a notable phenotypic switch, as shown by an elevated proportion of lipid-associated macrophages. However, APC maintenance was compromised during obesity development. We further validated that ATM-derived exosomal miR-690 is critical to maintain the APC population. In contrast, miR-690 abundance was greatly repressed in both CD9-and CD9+ ATMs in obesity, concomitant with lowering APC miR-690 abundance and population. Consistently, APC-specific miR-690 mutation led to a reduction in eWAT APC number in lean mice, further demonstrating the importance of miR-690 on APC maintenance. In addition, blocking macrophage recruitment by CCR2 knockout attenuated APC loss in obese adipose tissues. In obese fat pads, ATM-derived proinflammatory cytokines may also contribute to the depletion of miR-690 in APCs and impair their maintenance. Previous studies suggest that stem cells could exert profound regulation on neighboring macrophage activation [[49], [50], [51]], raising the possibility that APCs might influence ATM behavior, including ATM miR-690 expression.

NAD homeostasis is essential for stem cell maintenance and activation. Early studies have shown that NAD + metabolism mediates APC activation by regulating α-ketoglutarate levels, which contribute to the demethylation of histone H3K9 in the PPARg promoter [52]. Additionally, other studies have highlighted the importance of NAD + levels for adipose tissue function and overall metabolism, with reduced NAD + biosynthesis being associated with impaired preadipocyte differentiation and metabolic dysfunction in both white and brown adipose tissue [[53], [54], [55], [56], [57], [58]]. Our previous studies have demonstrated that miR-690 regulates the functions of several cell types by repressing NAD kinase abundance and subsequent NAD + homeostasis [26]. Nadk is crucial for converting NAD to NADP, which is required for fatty acid synthesis and lipid storage [59]. Consistently, we validated that *Nadk* abundance is inversely associated with miR-690 levels. More importantly, APC-specific *Nadk* mutation minimized the effect of obesity on APC maintenance in visceral adipose tissues, demonstrating the important role of *Nadk* in mediating APC phenotypes in response to obesity. An early study by Ratnayake et al., 2021 [48] suggested that macrophages secrete NAMPT, a key mediator of NAD biosynthesis, into the muscle stem cell niche in response to muscle injury. Thus, there is a possibility of the role of NAMPT in the crosstalk between ATMs and APCs.

Weight loss is closely associated with the reduction in adipose tissue mass and improvements in metabolic markers [47,60,61]. Previous studies have shown that while the total ATM population remains relatively unchanged in weight loss models, the levels of ATM-derived pro-inflammatory cytokines are significantly reduced in visceral fat pads [47]. Our study observed that weight loss was accompanied by recovery of miR-690 abundance in both ATMs and APCs. Indeed, our previous studies have demonstrated that miR-690 overexpression significantly repressed pro-inflammatory responses in macrophages. More importantly, restoring miR-690 is critical for an increase in the APC number in the eWAT of weight loss mice. Our previous studies have shown the RNA splicing factor SRSF3 (serine-rich splicing factor 3) as playing a key role in weight loss-induced embryonic Kupffer cell phenotypes [45]. The importance of SRSF3 on miR-690 expression of ATMs or APCs in response to weight loss could be further explored in future studies.

In summary, our findings highlight the significance of the miR-690-*Nadk* regulatory axis in mediating ATM regulation on APC maintenance and activation across lean, obese, and weight-loss conditions in visceral fat tissues. Thus, these studies suggest that targeting the miR-690-Nadk regulatory axis could be a new strategy for promoting healthy adipose tissue expansion.

## 4 Materials and methods

### 4.1 Animals

C57BL/6 (B6) mice were fed either a normal chow diet (Lab Diet) or a high-fat diet (HFD) containing 60% of calories from fat, 20% from protein, and 20% from carbohydrates (Research Diets). Both diets were provided *ad libitum*. For most experiments, 8-week-old mice were fed with HFD for 1, 2, 8, 12, or 18 weeks, depending on the specific study design. In diet-switch experiments, mice were fed the HFD for 14 weeks and then either continued on HFD or switched to a normal chow diet for an additional 4weeks. B6 wild-type (WT) mice, Rosa26-LSL-dCas9 (stock number #43926), LysMcre (stock number #004781), CCR2^−/−^ (CCR2KO, stock number #004999), Rosa26-LSL-Cas9 (stock number # 028551), and PDGFRβ-P2A-CreER^T2^ (stock number #030201) were purchased from the Jackson Laboratory. Nadk^flox/flox^ mice were purchased from Cyagen. All animals were acclimated to the housing conditions at the University of California, San Diego animal facility for 1 week before the start of experimental treatments and breeding. To generate macrophage- and adipocyte precursor cell (APC)-specific Cas9 transgenic mice, Rosa26-LSL-Cas9 knock-in mice were bred with LysMcre and PDGFRb-P2A-CreER^T2^ mice, respectively. APC-specific dCas9 transgenic mice were generated by crossing PDGFRb-P2A-CreER^T2^ with Rosa26-LSL-dCas9. Additionally, PDGFRb-P2A-CreER^T2^ mice were crossed with Nadk^flox/flox^ mice to generate an APC-specific knockout of *Nadk* (PDGFRbCre^+^Nadk^flox/flox^), with Nadk^flox/flox^ mice serving as controls. For the PDGFRb-P2A-CreER^T2^ mice, Cre activity was activated by intraperitoneal injections of tamoxifen (75 mg tamoxifen/kg body weight). For generating specific knockouts via Clustered Regularly Interspaced Short Palindromic Repeats (CRISPR)/CRISPR-associated protein 9 (Cas9) of Ybx1 and miR-690 in macrophages and APCs within the eWAT, LysMCre^+^Cas9^+^ and PDGFRbCre^+^Cas9^+^ mice were injected directly into one eWAT pad with gRNA targeting Ybx1 or miR-690, while the other pad was injected with lentivirus carrying empty vectors. The gRNAs were administered at a concentration of 10^7^ particles per mouse. Transcriptional activation of miR-690 using CRISPR/deactivated Cas9 (dCas9) was achieved by directly injecting one eWAT pad with gRNA targeting miR-690 along with the co-activator vP64 in PDGFRbCre^+^dCas9^+^ mice. All mice used in this study were male and maintained at 22 °C in a 12/12 h light–dark cycle in a specific pathogen-free facility and given free access to food and water.

### 4.2 Study approval

All animal procedures were done in accordance with University of California, San Diego Research Guidelines for the Care and Use of Laboratory Animals and all animals were randomly assigned to cohorts when used.

### 4.3 Direct eWAT injections

Mice were anesthetized with an intraperitoneal injection of ketamine (100 mg/kg) and xylazine (10 mg/kg), and placed on their back at the surgery station, with the abdomen exposed. The hair in the region where the epididymal white adipose tissue (eWAT) fat pads are expected (lateral edge on both sides of the lower abdominal region) was shaved, and the area was sterilized using iodine and 70% isopropyl alcohol pads. A small incision was made on one side of the eWAT pad, using surgical scissors, to open the skin. The peritoneum was not opened, as the eWAT fat pad was visible without doing so. Using a 0.5 mL insulin syringe, gRNAs (100-150uL), clodronate liposomes (∼70uL), and control PBS liposomes (∼70uL) were injected directly into the eWAT pad. The total injection volume was evenly divided and administered as 3–5 smaller injections at different sites within the eWAT to ensure uniform distribution throughout the tissue. After injection, the incision was sutured, and the same procedure was repeated for the other eWAT pad. To prevent post-operative infections, iodine was applied to the incision sites. For pain management, animals were treated topically with 4% lidocaine in the incision regions twice a day for 5 days following the surgery.

### 4.4 Macrophage depletion with clodronate liposomes

Clodronate liposomes (C-005, LIPOSOMA) were administered to lean WT B6 mice twice a week at a concentration of 0.35 mg/mouse (∼70uL), resulting in whole-body macrophage depletion, including in the eWAT, compared to animals treated with the same concentration of PBS control liposomes. In addition, lean WT B6 mice received direct injections of clodronate into one eWAT pad, while the other pad was injected with control PBS liposomes to specifically deplete macrophages in that region after 4 days of treatment.

### 4.5 Isolation of the stromal vascular cells from eWAT

eWAT was dissected and minced into small pieces using scissors. The minced tissue was then digested with collagenase IV (#C2139, Worthington) solution (1 mg/mL) for 20–40 min at 37 °C, shaking gently to ensure even digestion. Following digestion, the cell suspension was filtered using Nylon Biopsy Bags (#62324-35, Electron Microscopy Sciences) and centrifuged at 2000×*g* for 10 min at 4 °C. After centrifugation, the stromal vascular cells (SVC) pellet was obtained. The pellet was resuspended with Red Cell Lysis Buffer, washed, and then prepared for FACS staining or Matrigel implantation.

### 4.6 Flow cytometry analysis and sorting

The SVC pellet was resuspended in an Fc receptor blocker (anti-CD16/32) at a dilution of 1:200 for 30 min to minimize nonspecific binding. After this incubation, the cells were incubated with fluorescence-tagged antibodies for an additional 30 min. Following the antibody incubation, the cells were washed and prepared for flow cytometry analysis. The analysis involved sorting various populations, including adipose tissue macrophages (ATMs), characterized as CD45+ CD11B + F4/80+, as well as CD9+ ATMs (CD45+ CD11B + F4/80+ CD9+), CD9- ATMs (CD45+ CD11B + F4/80+ CD9-), and APC defined as PDGFRβ+ CD9- LY6C- [18,62]– Gating strategy is described in Figure 1B. To assess apoptosis, cells were stained using Annexin-FITC Apoptosis Detection Kit (#88-8005-72, ThermoFisher Scientific) after antibody staining for cell surface markers as described above. The sorting and analysis were conducted using the MA900 flow cytometer (Sony). Data were analyzed using FlowJo v10 software (BD Life Sciences).

### 4.7 EV isolation

Cells were cultured in media supplemented with extracellular vesicle (EV)-depleted fetal bovine serum (FBS; #A2720801, Thermo Fisher Scientific) for an additional 24 h. Conditioned media were collected and subjected to sequential centrifugation (400×*g* for 10 min, 2,000×*g* for 10 min, and 16,000×*g* for 40 min) followed by ultracentrifugation (120,000×*g* for 4 h at 4 °C) using an Optima XPN-80 ultracentrifuge (Beckman Coulter). Particle size and concentration were determined using a NanoSight NS300 with NTA software (v3.4, Malvern Instruments). RNA from EVs was extracted using the Total RNA Purification Kit (#42800, Norgen Biotek) according to the manufacturer's protocol. miR-690 levels in ATM-derived EVs were quantified by qPCR.

### 4.8 Culture, differentiation and transfection of 3T3-L1 adipocytes

3T3-L1 fibroblast cells (CL-173, ATCC) were cultured in Fibroblast Medium consisting of DMEM supplemented with 10% fetal bovine serum, 1% penicillin-streptomycin (#15140122, Gibco), and 1% GlutaMAX (#35050061, Gibco) at 37 °C in 5% CO_2_. For differentiation, cells were seeded in 24-well plates and allowed to reach confluence. One day after reaching 100% confluency, cells were transfected with either siRNA targeting Nadk (#J-053672-05-0002, Dharmacon), miR-690 mimic (#4464066, ThermoFisher Scientific), or a combination of both using Lipofectamine RNAiMAX (#13778030, ThermoFisher Scientific), following the manufacturer's protocol. A scrambled sequence was used as a negative control. After 24 h, the medium was replaced, and differentiation was initiated two days after reaching confluency. Adipocyte differentiation (day 0) was induced by replacing the medium with 1 mL per well of Differentiation Medium A (DMEM containing 10% FBS, 1 μg/mL insulin, 0.25 μM dexamethasone, and 5 μM IBMX) for 3 days. On day 3, the medium was replaced with Differentiation Medium B (DMEM containing 10% FBS and 1 μg/mL insulin), and cells were cultured for an additional 6 days with medium changes on days 3 and 5. On day 7, intracellular lipid accumulation was assessed.

### 4.9 LipidSpot staining and triglycerides quantification in 3T3-L1 adipocytes

At the end of the differentiation period, intracellular lipid accumulation in 3T3-L1 adipocytes was assessed by LipidSpot staining and triglyceride quantification. LipidSpot 488 Lipid Droplet Stain (#70065-T, Biotium) was used according to the manufacturer's instructions. Briefly, cells were washed with PBS and incubated with LipidSpot staining solution diluted 1:1000 in serum-free DMEM for 30 min at 37 °C, protected from light. After incubation, cells were washed again with PBS, and fluorescent images were acquired using a JuLI Stage fluorescence microscope (Nanoentek) with a 4 × objective lens. For lipid droplet quantification, at least three non-overlapping fields per well were captured from a minimum of three wells per treatment group. Lipid droplet size and number were analyzed using ImageJ software (NIH). Quantification parameters included droplet size distribution, mean droplet area, and the frequency of small (1–5 μm^2^) and large (20–80 μm^2^) lipid droplets. For triglyceride quantification, intracellular triglyceride content was measured using the Triglyceride Colorimetric Assay Kit (#NBP3-24540, Novus Biologicals) according to the manufacturer's instructions and normalized to total protein content determined by Bradford's Reagent (#E530-1L, VWR Chemicals).

### 4.10 Matrigel implant

The Matrigel implant procedure was adapted from Schwalie et al., 2018 [46]. Briefly, approximately 1 × 10^6^ cells from the SVC of WT and APC-NadkKO mice were resuspended in 400 μL of Matrigel (CB-40234, Corning) and injected subcutaneously, above the peritoneal cavity of the same lean WT young mice. Following the implantation, the animals were placed on a HFD for either 2 or 4 weeks. After this period, the Matrigel implants were either processed for FACS analysis, using the same collagenase digestion and antibody staining protocol described for eWAT, or prepared for morphology examination with hematoxylin and eosin (H&E) staining.

### 4.11 Quantitative RT–PCR analysis

Total RNA was isolated using either TRIzol (15596026, Invitrogen) or the Quick-RNA MicroPrep kit (11–328 M, Zymo Research). For mRNA analysis, reverse transcription was performed using the High-Capacity cDNA Reverse Transcription kit (4368813, Thermo Fisher Scientific), while reverse transcription for miRNA-690 was conducted using the TaqMan MicroRNA Reverse Transcription kit (4366597, Thermo Fisher Scientific). Quantitative PCR (qPCR) for mRNA was performed in a 10 μL reaction using PerfeCTa SYBR Green FastMix (95073-05 K, Quantabio VWR), whereas miRNA-690 was analyzed with Fast Advanced Master Mix (4444557, ThermoFisher Scientific) on a QuantStudio™ 3 Real-Time PCR System (Thermo Fisher Scientific). The data, presented as the mean of 2-ΔΔCt, were normalized to the housekeeping gene Actb for mRNA and to the endogenous control U6 for miRNA.

### 4.12 eWAT histology

eWAT and Matrigel-containing SVC cells were fixed in 10% neutral buffered formalin for 24 h at room temperature. Following fixation, the samples were embedded in paraffin and sectioned into 5 μm-thick slices using a microtome, which were then placed on glass slides. After deparaffinization, the sections underwent hematoxylin and eosin staining. Finally, the stained slides were scanned using the VS200 Slide Scanner (Olympus). Different regions of the same eWAT depot were analyzed. Five representative photos were taken of each section, and adipocyte diameter was measured with ImageJ/Fiji (NIH) software as previously described [63].

### 4.13 eWAT immunofluorescence

Paraffin-embedded eWAT sections were deparaffinized in xylene (2 × 5 min) and rehydrated through a graded ethanol series (100%, 95%, and 70%) to distilled water. Antigen retrieval was performed by incubating slides in citrate buffer (10 mM sodium citrate, pH 6.0) at 95–100 °C for 10 min, followed by a 30-minute cool down at room temperature. Slides were then washed in PBS and permeabilized with 0.3% Triton X-100 in PBS (PBS-T) for 15 min. Blocking was carried out in 5% normal goat serum (NGS) in PBS-T for 1 h at room temperature. Sections were incubated overnight at 4 °C with the following primary antibodies diluted in 1% NGS in PBS-T: anti-pAKT (Cell Signaling Technology, #40605, 1:400) and anti-PLIN1 (Thermo Fisher Scientific, MA5-27861, 1:200). After washing in PBS, sections were incubated with appropriate secondary antibodies: goat anti-mouse conjugated to Cy5 (Thermo Fisher Scientific, #A10524) and goat anti-rabbit conjugated to Alexa Fluor 488 (Thermo Fisher Scientific, #A11008), for 2 h at room temperature in the dark. Nuclei were counterstained with DAPI (1 μg/mL) for 10 min. Slides were mounted with ProLong™ Gold Antifade Mountant (Thermo Fisher Scientific) and imaged using a Keyence fluorescence microscope. Imaging parameters were kept constant across all samples. Quantification of fluorescence intensity and adipocyte morphology was performed using ImageJ/Fiji (NIH) software on at least three fields per section, with four biological replicates per group.

### 4.14 Single-nucleus RNA sequencing datasets and data processing

snRNA-seq datasets for mouse and human visceral adipose tissue were obtained from the Gene Expression Omnibus (GEO) databases GSE160729 and GSE176067. Processed data were available for download as Seurat objects, which were used as the basis for subsequent analyses. Clustering was performed following the protocols outlined by the original authors. The R package Seurat (version 5.0.2) [64] was employed to identify LAM and APC populations and to evaluate the expression of key genes, including *Trem2*, *Cd9*, *Gpnmb*, *Adgr1*, and *Nadk*, and survival markers.

### 4.15 Statistical analysis

Mice were randomly assigned to experimental groups for all in vivo studies. Statistical tests used for data analysis are detailed in the respective figure legends. An unpaired two-tailed Student's t-test was used to compare the means between the two groups. All statistical analyses were performed using Prism 8 software (version 8.0; GraphPad, La Jolla, CA). A p-value of ≤0.05 was considered statistically significant, with the specific degrees of significance indicated in the figure legends.

## CRediT authorship contribution statement

**Karina Cunha e Rocha:** Writing – original draft, Methodology, Formal analysis, Data curation. **Breanna Tan:** Validation, Data curation. **Julia Kempf:** Validation, Data curation. **Cristina Medina:** Data curation. **Varsha Beldona:** Data curation. **Chengjia Qian:** Data curation. **Ying Duan:** Data curation. **Qian Xiang:** Data curation. **Ahjin Yoo:** Data curation. **Xiaomi Du:** Visualization, Data curation. **Amit R. Majithia:** Writing – review & editing, Validation, Data curation. **Wei Ying:** Writing – review & editing, Supervision, Resources, Project administration, Funding acquisition, Formal analysis.

## Funding

This study was funded by National Institutes of Health grants (R21HD107516, R00DK115998, and R01DK125560 to W.Y. and U24DK132746-01 to K.C.R.) and by the Larry L. Hillblom Foundation (2023-D-011-FEL to K.C.R.).

## Declaration of competing interest

The authors have nothing to declare.

## Data Availability

Data will be made available on request.

## Appendix A Supplementary data

Multimedia component 1 **Fig. S1Effect of adipose tissue macrophages on adipocyte precursor cell maintenance.** (**A**) Adipose tissue macrophages **(**ATMs) population in epididymal white adipose tissue (eWAT) after 2 weeks of clodronate liposome treatment. (**B**) Gating strategy for adipocyte precursor cells (APCs) in eWAT. FACS analysis of PKH26-labeled (**C**) clodronate liposome or (**D**) lentivirus to assess potential leakage following local injection into eWAT. (**E**) ATM population in eWAT after local injection of lentivirus carrying gRNA-miR690. (**F**) Ybx1 expression in ATMs confirming knockdown efficiency. (**G**) miR-690 copy number per EV particle was analyzed by qPCR. ATM-derived EVs were isolated from the conditional media, and their **(H)** particle concentration, and **(I)** size were assessed. Data are presented as mean ± SEM. ^∗^*P* ≤ 0.05, ^∗∗^*P* ≤ 0.01, ns = not significant. Student's t-test. *n* = 4 mice/group for (**A**), *n* = 3 mice/group for (**C–D**), *n* = 13 (control) and 14 (miR-690 gRNA) for (**E**), and *n* = 3 mice/group for (**F–G**). **Fig. S2. Lipid-associated macrophage phenotypes in response to obesity. Expression of lipid-associated macrophage** (LAM) marker genes in (**A**) human or (**B**) mouse visceral fat tissue by snRNAseq analysis, respectively. (**C**) FACS analysis of CD9+ adipose tissue macrophages (ATMs) in the epididymal white adipose tissue (eWAT) of lean vs. 12 weeks HFD-fed mice. (**D**) Total ATM population and (**E**) proportion of CD9+ ATMs in the eWAT after 7 days of HFD feeding. Data are presented as mean ± SEM. ^∗∗∗^*P* ≤ 0.001, Student's t-test. *n* = 10 individuals total for (**A**); *n* = 7/group for (**B**); *n* = 4 (NCD WT) and *n* = 5 (12-week HFD WT) for (**C**); *n* = 5 mice/group for (**D–E**). **Fig. S3. eWAT phenotypes in CCR2KO after under normal chow and high-fat diet feeding.** Twenty-five-week-old CCR2KO mice and age-matched WT controls were assessed under normal chow diet (NCD) feeding. (**A**) Body weight, (**B**) liver weight, and (**C**) epididymal white adipose tissue (eWAT) weight were measured. FACS analysis of adipose tissue macrophage (ATM) populations in eWAT, including (**D**) total ATMs, (**E**) CD9+ATMs, and (**F**) CD9- ATMs. (**G**) FACS analysis of the adipocyte precursor cell (APC) population. (H) Histological analysis of eWAT showing adipocyte area. After 2 weeks of high-fat diet (HFD) feeding, (**I**) body weight, (**J**) epididymal white adipose tissue (eWAT) weight, and (**K**) eWAT adipose tissue macrophage (ATM) population were assessed in WT vs. CCR2KO mice. Effects of 8 weeks of HFD feeding on (**L**) body weight, (**M**) eWAT weight, (**N**) liver weight, and (**O**) eWAT ATM population in WT and CCR2KO mice. Data are presented as mean ± SEM. ^∗^*P* ≤ 0.05, ^∗∗^*P* ≤ 0.01, Student's t-test. *n* = 3 (NCD WT) and *n* = 4 (NCD CCR2KO) for (**A–H**); *n* = 11 (2-week HFD WT) and *n* = 13–14 (2-week HFD CCR2KO) for (**I–K**); *n* = 5 (8-week HFD WT) and *n* = 4 (8-week HFD CCR2KO) for (**L–O**). **Fig. S4. Effect of miR-690 abundance on adipocyte precursor cell phenotypes**. (**A**) Gene markers identifying adipocyte precursor cells (APCs) in human visceral fat. (**B**) miR-690 deletion efficiency in APCs of lean PDGFRbCre^+^Cas9^+^ mice following eWAT injection of lentivirus carrying gRNA-miR690. Effects of 5 weeks of miR-690 mimic intraperitoneal injections during HFD feeding on (**C**) body weight, and (**D**) eWAT weight in WT mice. **(E)** Representative immunofluorescence images (n = 4 eWAT samples) from 8-week high-fat diet (HFD)-fed PDGFRbCre^+^dCas9^+^ mice after unilateral eWAT injections of lentivirus expressing miR-690gRNA/VP64 (miR-690oe) or VP64 alone (control). Quantification of relative **(F)** pAKT and **(G)** PLIN1 fluorescence intensity. Data are presented as mean ± SEM. ^∗^*P* ≤ 0.05, Student's t-test. Scale bar, 100 μm. *n* = 10 individuals total for (**A**); *n* = 5 mice/group for (**B**); *n* = 7 (NCD WT), *n* = 6 (HFD WT + Scrambled mimic), and *n* = 5–6 (HFD WT + miR-690 mimic) for (**C**); *n* = 5 (5wks HFD WT) and *n* = 6 (HFD + miR-690 mimic) for (**D**); *n* = 4 mice/group for (**F–G**). **Fig. S5. miR-690 Protects APCs from Obesity-Induced Apoptosis.** UMAP visualization of **(A)***Mki67* and **(B)***Bcl2* expression across adipose stem and progenitor cell (ASPC) populations from mouse eWAT. **(C)** Expression levels of *Mki67* and *Bcl2* in the APC populations (ASPC1 and ASPC6). Average expression and percentage of cells expressing **(D)***Bcl2*, **(E)***Bak1*, and **(F)***Cycs* in APC populations from lean and HFD-fed mice. **(G)** Percentage of Annexin V + APCs after direct injection of control mimic or miR-690 mimic–containing liposomes into the eWAT of lean wild-type mice, followed by 1 week of HFD feeding. Statistical analysis was performed using Welch's two-sample t-test for (**D–F**) and Student's t-test for (**G**). *P*-values for (**D–F**) are displayed in the figure, with *P* ≤ 0.05 considered statistically significant. Data in (**G**) are presented as mean ± SEM with ^∗^*P* ≤ 0.05. *n* = 7 mice/group for (**A–F**); *n* = 4 mice/group for (**G**). **Fig. S6. Effect of NADK knockout on adipocyte precursor cell phenotypes**. (**A**) Expression of mature adipocyte marker expression in 6 clusters of Adipose Stem and Progenitor (ASPC) cells in mouse adipose tissue. After 8 weeks of high-fat diet feeding (HFD) feeding, (**B**) *Nadk* expression in adipocyte precursor cell (APC), (**C**) body weight, (**D**) weight gain, and (**E**) epididymal white adipose tissue (eWAT) weight were assessed in Nadk^f^^lox^^/f^^lox^ vs. PDGFRbCre^+^Nadk^f^^lox^^/f^^lox^ mice. (**F**) Assessment of mature adipocyte contamination in the stromal vascular cells (SVCs) embedded in Matrigel, collected 24 h after implantation. (**G**) Representative images of 3T3-L1 cells transfected with scrambled RNA (control), miR-690 mimic, siRNA-NADK, or a combination of miR-690 mimic and siRNA-NADK, differentiated into adipocytes for 7 days, and stained with LipidSpot to visualize neutral lipid accumulation. Quantification of (**H**) mean lipid droplet area, (**I**) frequency of large lipid droplets (20–80 μm^2^), (**J**) frequency of small lipid droplets (1–5 μm^2^), and (**K**) triglyceride content. Scale bars: (**F**) 1 mm, (**G**) 50 μm. Data are presented as mean ± SEM. ^∗^P ≤ 0.05, ^∗∗^P ≤ 0.01, ^∗∗∗^P ≤ 0.001, ^∗∗∗∗^P ≤ 0.0001. Student's t-test was used for panels **B–E**; one-way ANOVA followed by Sidak's post hoc test was used for panels **H–K**. *n* = 7 mice/group for (**A**); *n* = 4 mice/group for (**B**); *n* = 6 (8wks HFD *Nadk*^f^^lox^^/f^^lox^) and *n* = 10 (8wks HFD PDGFRbCre^+^*Nadk*^f^^lox^^/f^^lox^) for (**C–E**); *n* = 3 for (**F**); *n* = 7 wells/group for (**G–J**); *n* = 4–6 wells/group for (**K**). **Fig. S7****. Effect of weight loss on epididymal white adipose tissue phenotypes.** (**A**) Body weight and (**B**) eWAT weight after 4 weeks of diet-switch (DS) from a high-fat diet (HFD) to a normal chow diet (NCD). (**C**) Total weight loss, and populations of (**D**) CD9+ and (**E**) CD9-adipose tissue macrophage (ATM) in the epididymal white adipose tissue of WT vs. macrophage-specific miR-690 deletion mice. Data are presented as mean ± SEM. ^∗∗∗^*P* ≤ 0.001, Student's t-test. *n* = 5 (NCD WT control), *n* = 6 (18wks HFD WT), and *n* = 15 (4wks DS WT) for (**A**); *n* = 5 (NCD WT), *n* = 4 (18wks HFD WT), and *n* = 5 (4wks HFD DS WT) for (**B**); *n* = 3 (4wks DS WT) and *n* = 8–9 (4wks DS miR-690^▲ATMs^) for (**C–E**). Multimedia component 1
