# Supplementary figures and images for: Adipose tissue macrophage-derived miR-690 modulates adipocyte precursor cell maintenance and adipogenesis
Source: Mol Metab. 2025 Sep 3;101:102246. doi: 10.1016/j.molmet.2025.102246 (PMC12464593; doi:10.1016/j.molmet.2025.102246)

Figure S1

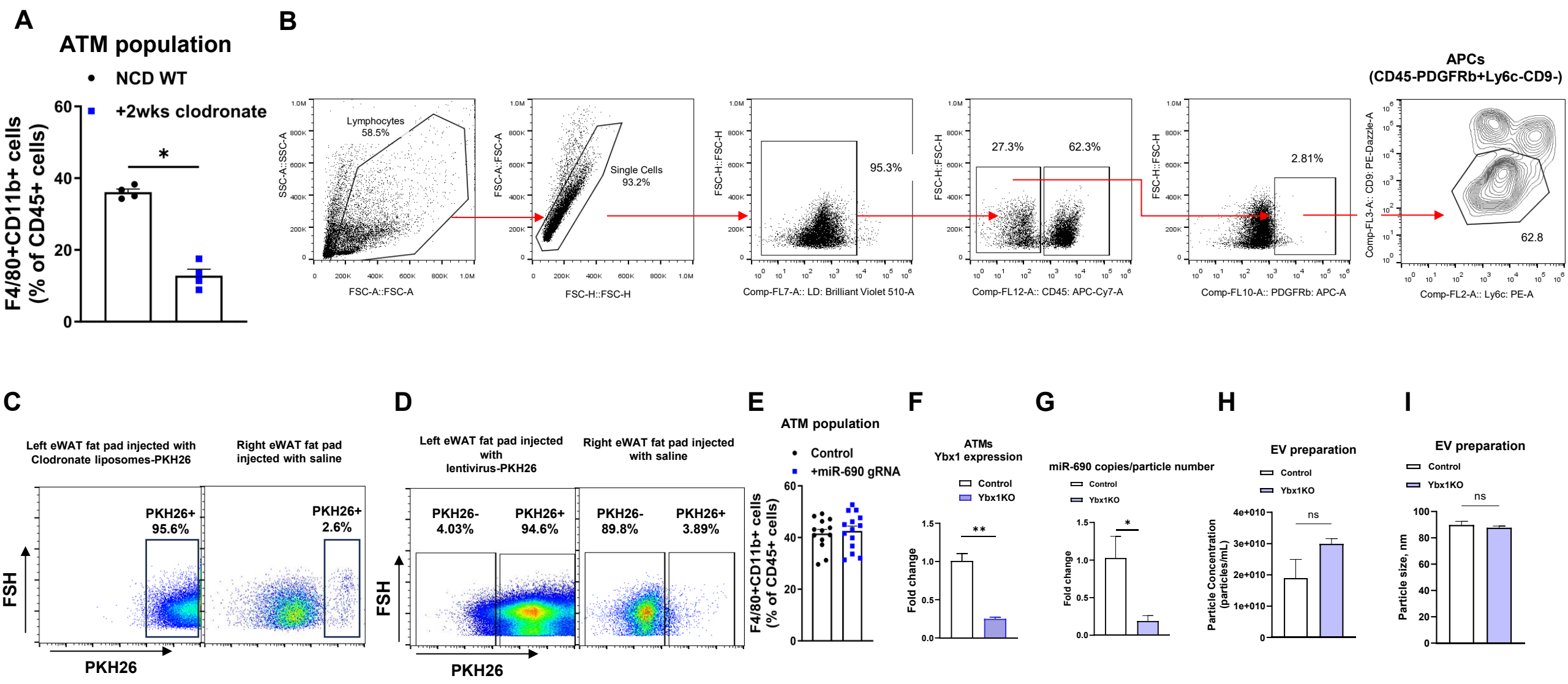

Figure S2

A

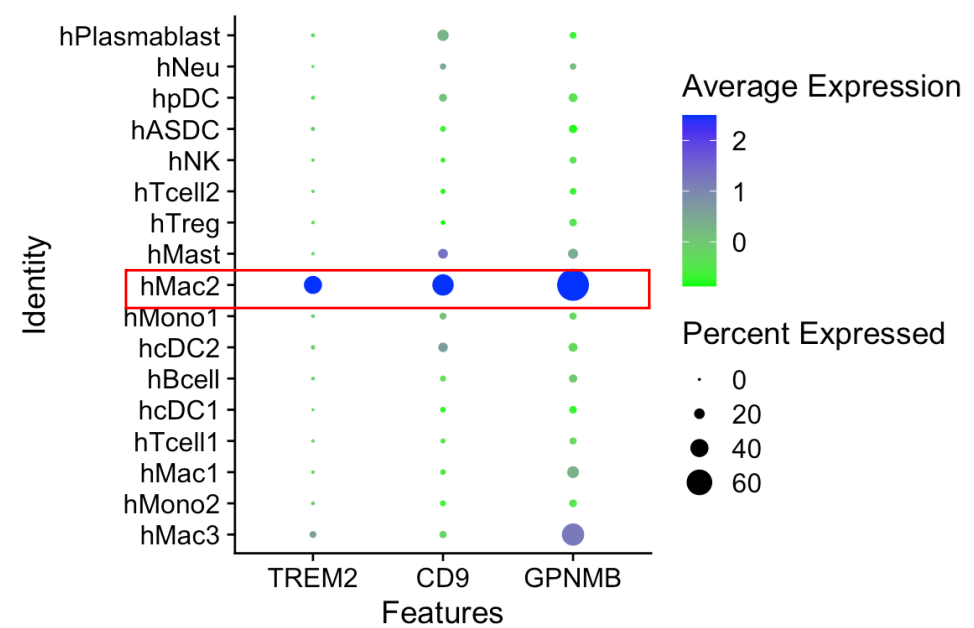

B

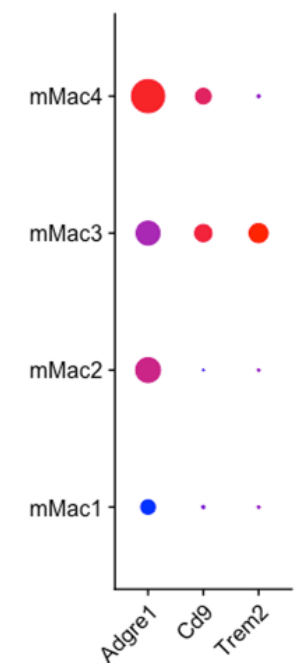

C

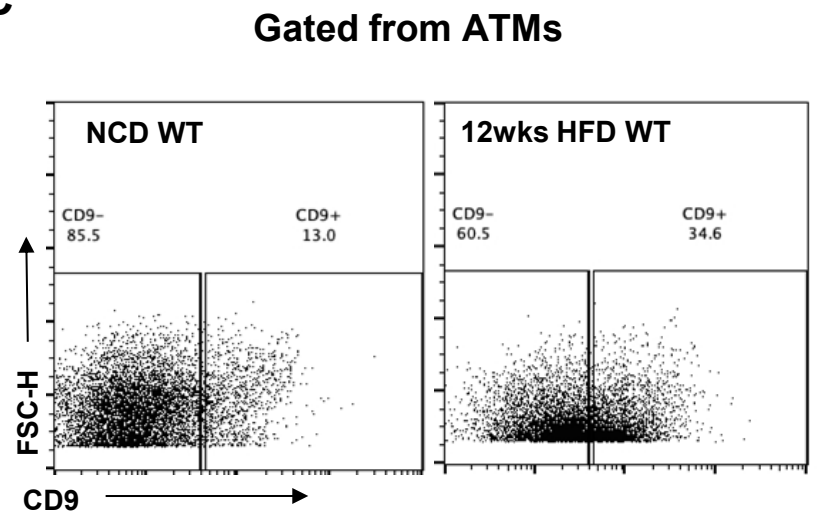

CD9+ ATMs

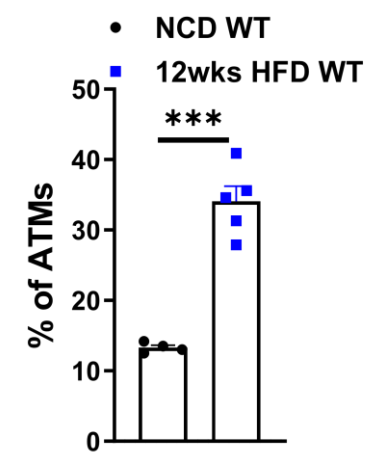

D

ATM population

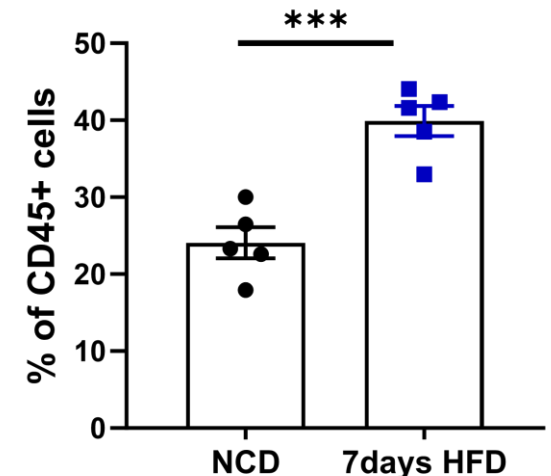

E

CD9+ ATMs

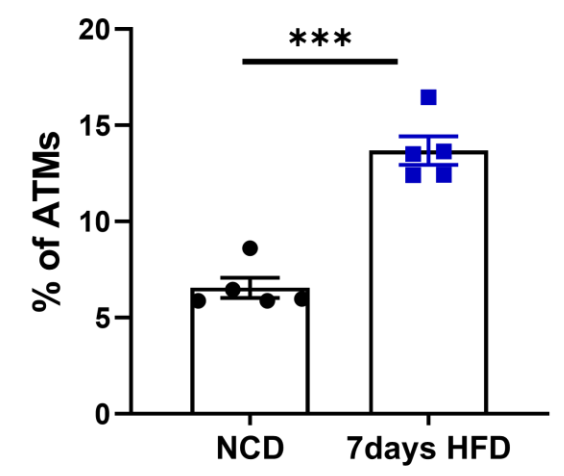

Figure S3

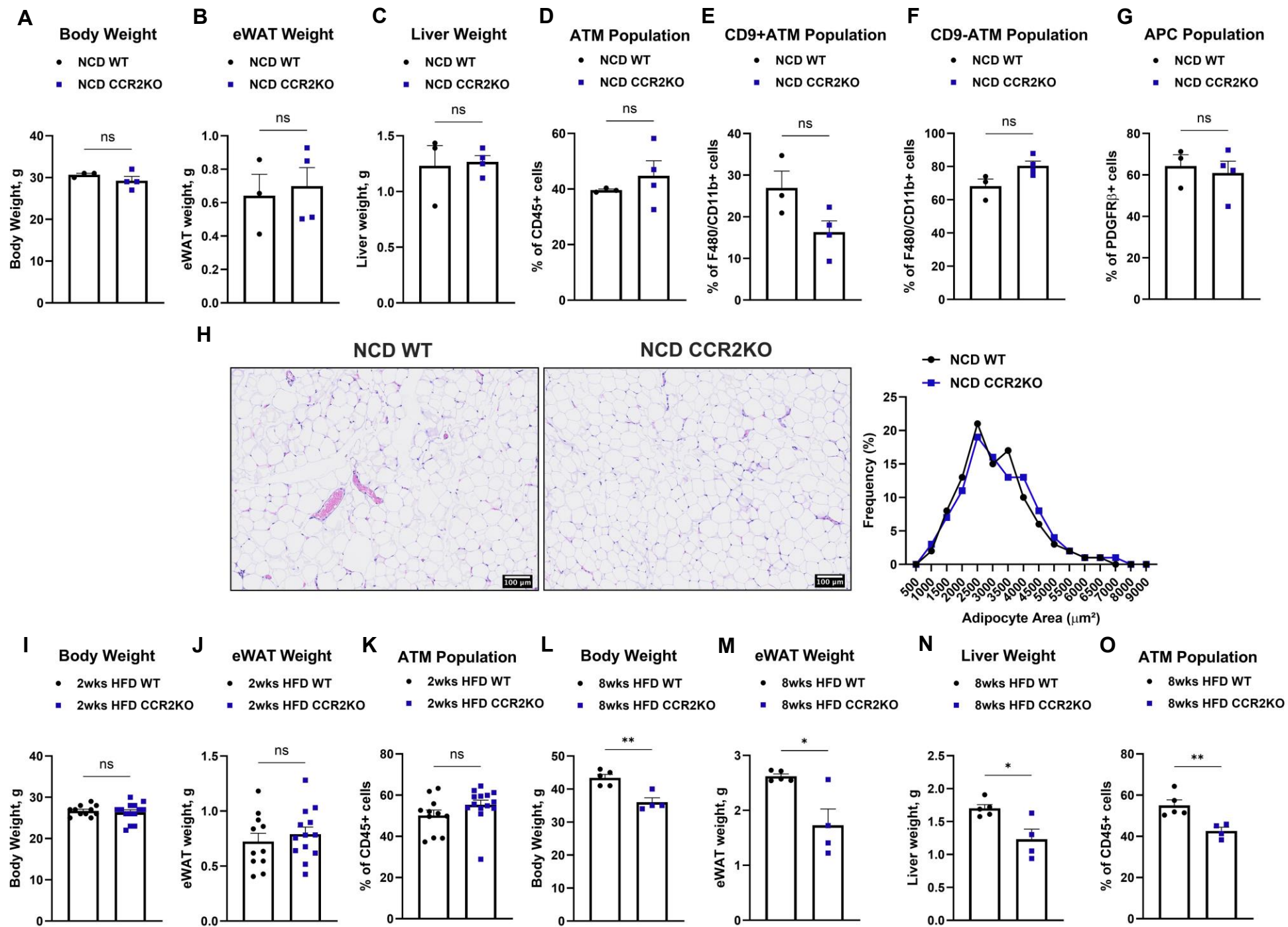

**Figure S4**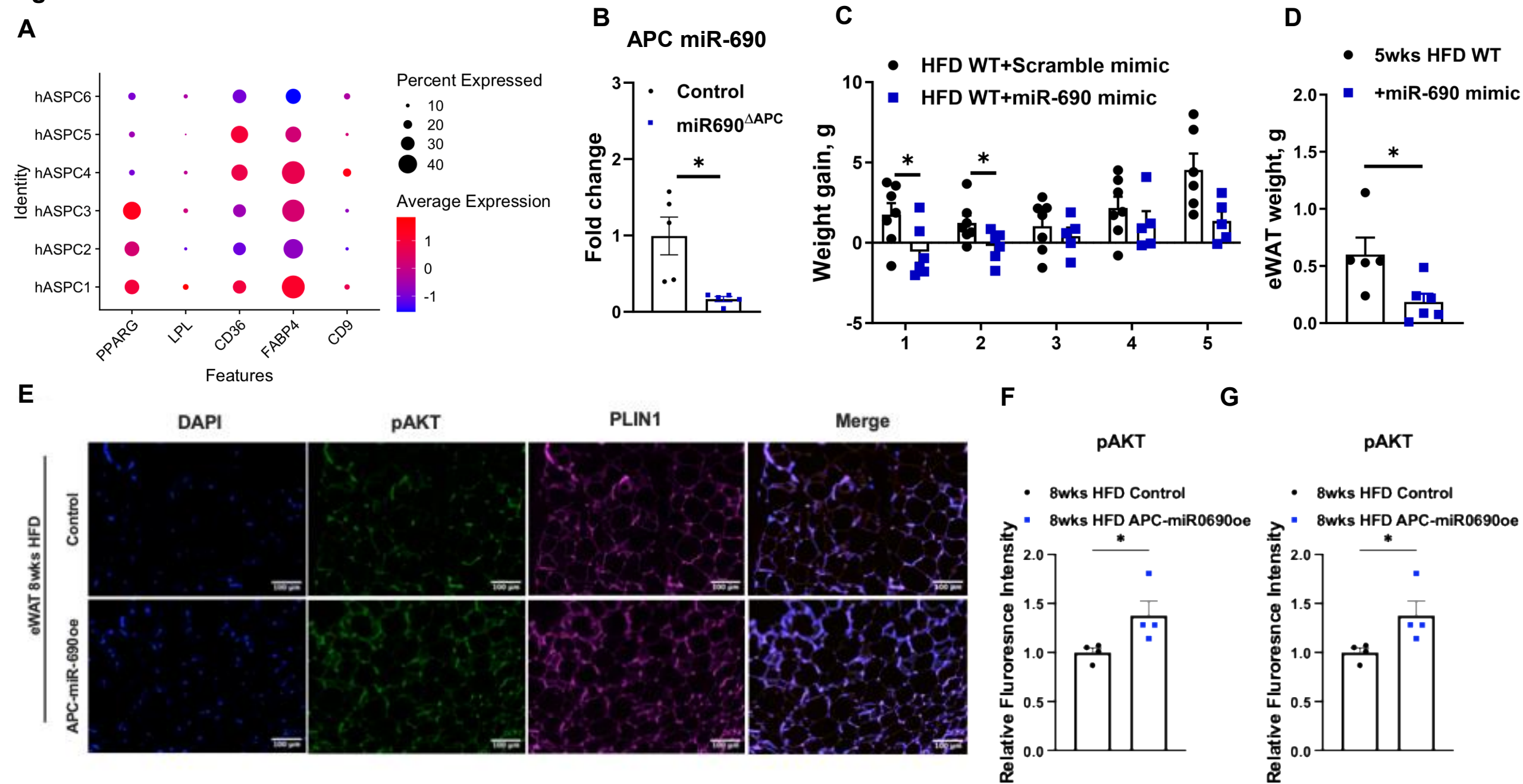

Figure S5

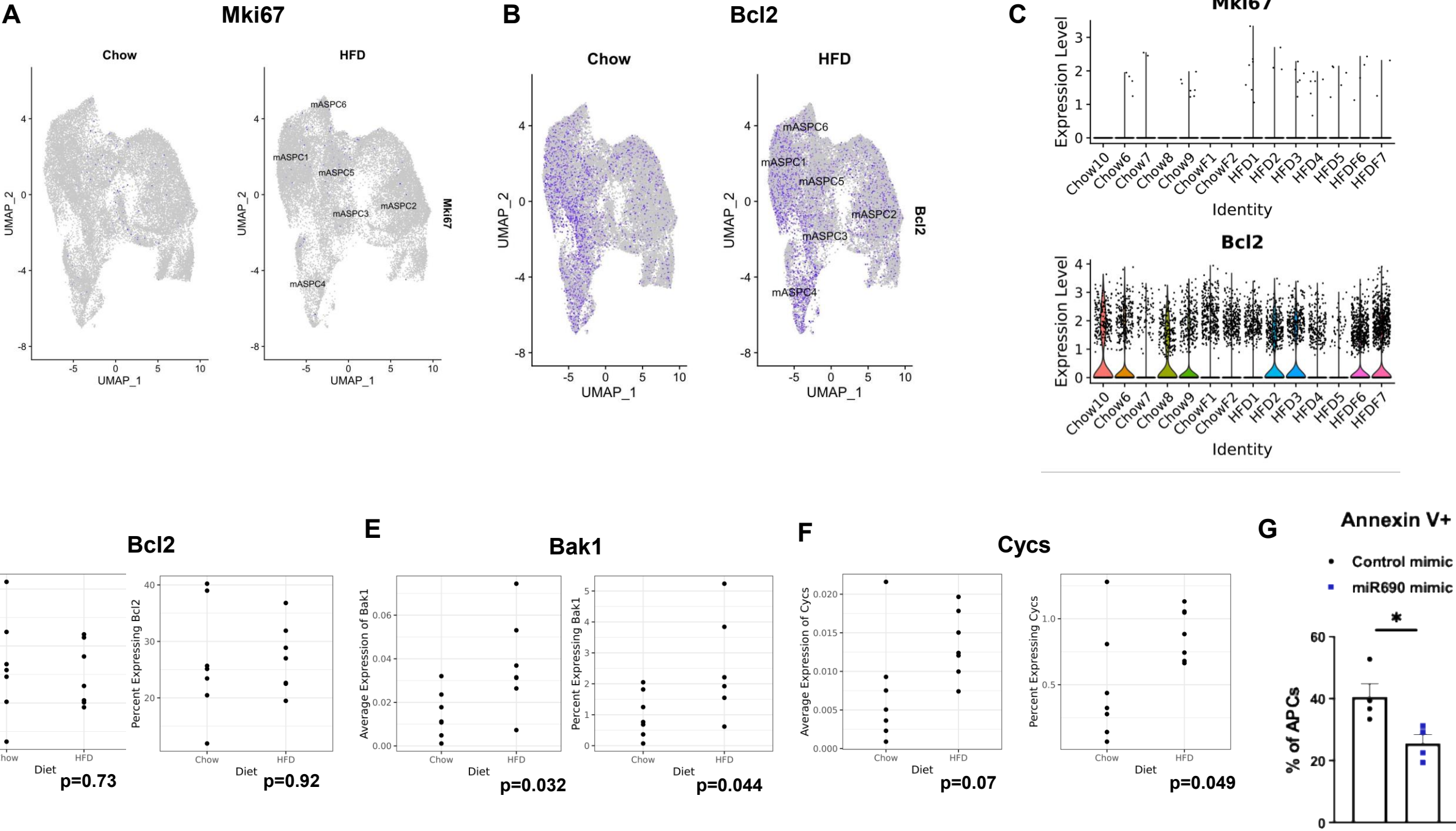

Figure S6

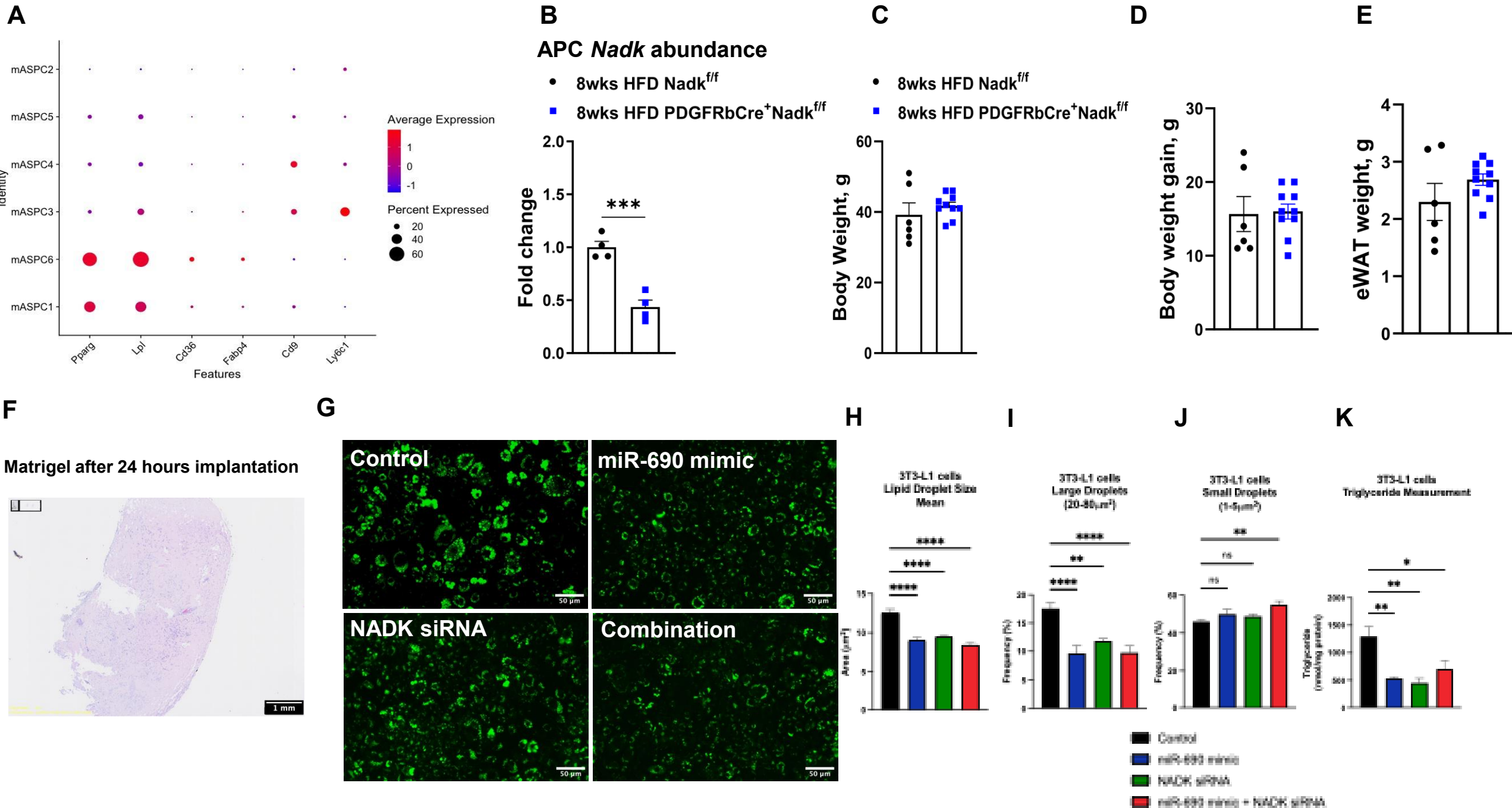

Figure S7

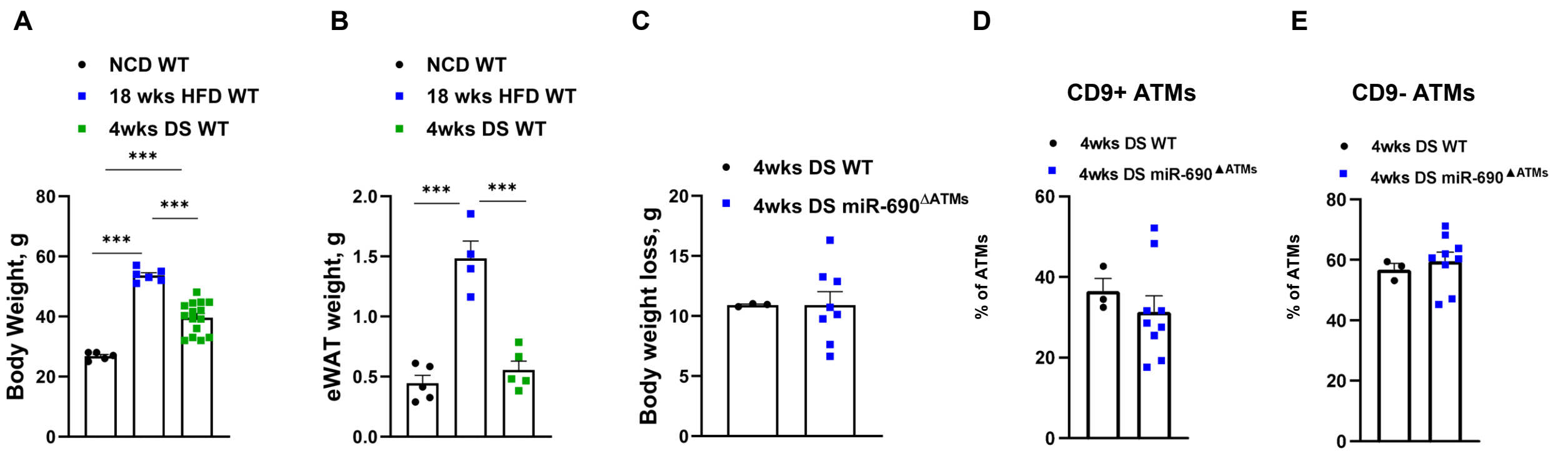

Supplement: Multimedia component 1 — Fig. S1Effect of adipose tissue macrophages on adipocyte precursor cell maintenance. (A) Adipose tissue macrophages (ATMs) population in epididymal white adipose tissue (eWAT) after 2 weeks of clodronate liposome treatment. (B) Gating strategy for adipocyte precursor cells (APCs) in eWAT. FACS analysis of PKH26-labeled (C) clodronate liposome or (D) lentivirus to assess potential leakage following local injection into eWAT. (E) ATM population in eWAT after local injection of lentivirus carrying gRNA-miR690. (F) Ybx1 expression in ATMs confirming knockdown efficiency. (G) miR-690 copy number per EV particle was analyzed by qPCR. ATM-derived EVs were isolated from the conditional media, and their (H) particle concentration, and (I) size were assessed. Data are presented as mean ± SEM. ∗P ≤ 0.05, ∗∗P ≤ 0.01, ns = not significant. Student's t-test. n = 4 mice/group for (A), n = 3 mice/group for (C–D), n = 13 (control) and 14 (miR-690 gRNA) for (E), and n = 3 mice/group for (F–G). Fig. S2. Lipid-associated macrophage phenotypes in response to obesity. Expression of lipid-associated macrophage (LAM) marker genes in (A) human or (B) mouse visceral fat tissue by snRNAseq analysis, respectively. (C) FACS analysis of CD9+ adipose tissue macrophages (ATMs) in the epididymal white adipose tissue (eWAT) of lean vs. 12 weeks HFD-fed mice. (D) Total ATM population and (E) proportion of CD9+ ATMs in the eWAT after 7 days of HFD feeding. Data are presented as mean ± SEM. ∗∗∗P ≤ 0.001, Student's t-test. n = 10 individuals total for (A); n = 7/group for (B); n = 4 (NCD WT) and n = 5 (12-week HFD WT) for (C); n = 5 mice/group for (D–E). Fig. S3. eWAT phenotypes in CCR2KO after under normal chow and high-fat diet feeding. Twenty-five-week-old CCR2KO mice and age-matched WT controls were assessed under normal chow diet (NCD) feeding. (A) Body weight, (B) liver weight, and (C) epididymal white adipose tissue (eWAT) weight were measured. FACS analysis of adipose tissue macrophage (ATM) pop [file mmc1.pdf]
